# Omega-3 Long-Chain Polyunsaturated Fatty Acids, EPA and DHA: Bridging the Gap between Supply and Demand

**DOI:** 10.3390/nu11010089

**Published:** 2019-01-04

**Authors:** Douglas R Tocher, Monica B Betancor, Matthew Sprague, Rolf E Olsen, Johnathan A Napier

**Affiliations:** 1 Institute of Aquaculture, Faculty of Natural Sciences, University of Stirling, Stirling FK9 4LA, UK; m.b.betancor@stir.ac.uk (M.B.B.); matthew.sprague@stir.ac.uk (M.S.); 2 Norwegian University of Science and Technology, Department of Biology, 7491 Trondheim, Norway; rolf.e.olsen@ntnu.no; 3 Department of Plant Sciences, Rothamsted Research, Harpenden AL5 2JQ, UK; johnathan.napier@rothamsted.ac.uk

**Keywords:** eicosapentaenoic acid, docosahexaenoic acid, novel sources, microalgae, oilseed crops, genetic modification, aquaculture

## Abstract

The omega-3 (n-3) long-chain polyunsaturated fatty acids (LC-PUFA), eicosapentaenoic (EPA, 20:5n-3) and docosahexaenoic (DHA, 22:6n-3) acids, are well accepted as being essential components of a healthy, balanced diet, having beneficial effects on development and in mitigating a range of pathological conditions. However, their global supply from all the traditional sources of these nutrients is insufficient to satisfy human nutritional requirements. For two decades there has been considerable research carried out into all possible alternatives to the main sources of n-3 LC-PUFA, marine fish oil and fishmeal, driven largely by the aquaculture sector, as both the major user and provider of EPA and DHA. In the last few years these efforts have focused increasingly on the development of entirely new supplies of n-3 LC-PUFA produced *de novo*. Recently, this has resulted in various new sources of EPA and/or DHA that are already available or likely to available in the near future. In this short review, we briefly summaries the current gap between supply and demand of EPA and DHA for human requirements, the role of aquaculture in providing n-3 LC-PUFA to human consumers, the range of potential novel sources, and suggest how these new products could be used effectively. We conclude that all the new sources have potentially important roles to play in increasing the supply of n-3 LC-PUFA so that they are available more widely and in higher concentrations providing more options and opportunities for human consumers to obtain sufficient EPA and DHA to support more healthy, balanced diets.

## 1. Introduction

**Figure 1:**
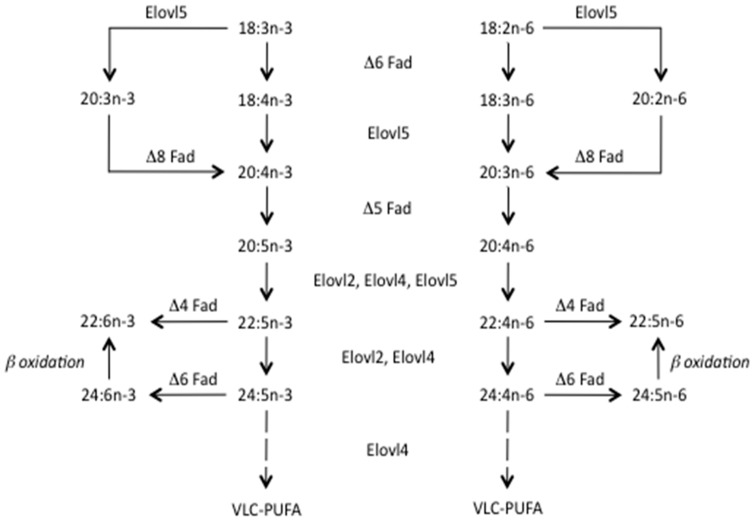
Pathways of long-chain polyunsaturated fatty acid biosynthesis in humans and fish. All activities other than Δ4 desaturation (Δ4 Fad) are present in humans. Similarly, all activities have been demonstrated in teleost fish species, although not all species express all activities. The presence of Δ4 Fad enabling direct production of 22:6n-3 from 22:5n-3 has only been demonstrated in a few teleost fish species and, therefore, DHA (22:6n-3) production from EPA (20:5n-3) in most fish species and humans is only possible via the Sprecher shunt [1]. Δ4 Fad, Δ5 Fad and Δ6 Fad, fatty acyl desaturases; DHA, docosahexaenoic acid; EPA, eicosapentaenoic acid; Elovl2, Elovl4 and Elovl5, fatty acid elongases. Reproduced by permission from Tocher [2].

While the omega-3 (n-3) long-chain polyunsaturated fatty acids (LC-PUFA), eicosapentaenoic (EPA, 20:5n-3) and docosahexaenoic (DHA, 22:6n-3) acids, can be produced endogenously by humans and some fish species [1], their rate of biosynthesis is low and insufficient to meet physiological demands [1,2] (Figure 1). Therefore, EPA and DHA are well accepted as being essential components of a healthy, balanced diet, having beneficial effects on development especially of the neural system [3,4], and in mitigating a number of pathological conditions [5,6,7]. However, what is not so well known or appreciated is that these nutrients are in short supply [8]. There is simply not enough available from all the traditional sources to satisfy human nutritional requirements, and aquaculture, both as a major user and provider of EPA and DHA, has been at the forefront in highlighting this gap between demand and supply, and driving the omega-3 research agenda [2]. In recent years, this has led to considerable academic and industry interest in developing entirely new supplies of n-3 LC-PUFA and this has resulted in various products that are already in production or likely to go forward to commercial production [9]. The present article briefly summarises the reasons behind the current gap in supply and demand, the role of aquaculture, the new products likely to be available commercially in the near future and longer term, and the opportunities and options these entirely novel sources of n-3 LC-PUFA can provide in helping to promote balanced and healthy human diets.

## 2. The Problem with the Available Supply of n-3 LC-PUFA

### 2.1. Gap between Supply and Demand

Our understanding of the beneficial effects of dietary EPA and DHA on human health has been based largely on three main lines of evidence including epidemiological studies, randomized controlled trials (RCT), and laboratory studies investigating biochemical and molecular mechanisms that have provided considerable mechanistic support to the in vivo approaches [7,10,11]. Based on all the evidence, many recommendations for EPA and DHA intake for a healthy human diet have been produced by a large number of global and national health agencies and associations, and government bodies [12,13]. The recommendations of over 50 organizations were compiled by the Global Organization for EPA and DHA Omega 3 s [14] and, based on the most commonly recommended dose for cardiac health (500 mg/person/day), the total demand for n-3 LC-PUFA can easily be calculated (500 mg/day × 365 days × 7 billion) to amount to over 1.27 million tonnes [2]. In contrast to the considerable research into the benefits of dietary n-3 LC-PUFA, EPA and DHA, on human health, and the amelioration of several pathological conditions [3,4,5,6,7,10,11,12], which has resulted in the plethora of dietary recommendations [13,14], few studies on humans have even considered where all the n-3 LC-PUFA would come from if the dietary recommendations were followed by the human population. However, three studies have attempted to calculate the difference between the demand to satisfy human physiological requirements and the actual available supply of EPA and DHA [2,8,15]. While the calculation of human requirements (demand) is relatively simple, calculating the available supply of n-3 LC-PUFA is not, and requires various assumptions and estimates [see 2]. However, despite this, all three studies came to the same conclusion, that the available supply of n-3 LC-PUFA was insufficient to meet the demand to satisfy human requirements [2,8,15]. Indeed, in the journal issue, which was dedicated to n-3 LC-PUFA and that contained the latest study to calculate n-3 LC-PUFA supply [15], the editors chose to highlight the question “where will all the omega-3 fatty acids come from” in the journal editorial [16]. Therefore, while the supply of n-3 LC-PUFA can be optimistically estimated at just over 0.8 million tonnes indicating a shortfall of more than 0.4 million tonnes [2] or, more pessimistically the deficit in supply has been calculated at over 1 million tonnes [15], the fact that a significant gap exists is not in question irrespective of how it is calculated [8]. Furthermore, the relentless increase in the global population translates into ever-growing demand.

### 2.2. Why Is There a Gap and What Impact Does It Have?

The calculations of supply in Tocher [2] highlight that the limitation in availability of EPA and DHA is linked to their aquatic, predominantly marine, origin as oceans accounts for 96.5% of all water on earth. While a very recent study has revealed that n-3 PUFA can be synthesised *de novo* in some marine invertebrates [17], the vast majority is produced at the base of the marine food web in marine microbes, including predominantly microalgae [18], whereas terrestrial plants do not produce EPA or DHA [19]. Consequently, our supplies of n-3 LC-PUFA come from the oceans, the majority (almost 90%) from capture fisheries, whether as food fish or via fish oil and fishmeal, with relatively small additional amounts estimated from seafood by-products and recycling, unfed aquaculture and traditional macroalgal sources [2]. Therefore, while other animal-derived foods can contribute small amounts of n-3 LC-PUFA [20,21], access to, and consumption of, fish and seafood is the major factor in determining how the diet of individuals and populations around the globe can satisfy n-3 LC-PUFA requirements. Indeed, a recent global survey of blood levels of EPA and DHA reported that “populations living on coastal regions of countries, and populations that traditionally rely on hunting, fishing and gathering for sustenance tended to have moderate to high blood levels of EPA+DHA” and concluded that, while there was considerable variability in blood levels of EPA+DHA, “the very low to low range of blood EPA+DHA for most of the world may increase global risk for chronic disease” [22]. In addition, public perceptions of fish, especially oily fish, leading to consumer resistance [21], as well and various lifestyle choices [23,24] can also impact intake of n-3 LC-PUFA. In consequence, a very large global study into consumption of dietary fats including 266 country-specific surveys, showed that the global mean consumption of n-3 LC-PUFA was 163 mg/day [25]. While there was variation in n-3 LC-PUFA consumption at both regional and national levels [25], the mean value was well below the lowest recommended level for intake of around 250 mg/day [13,14]. It is in this context that the fundamental, global lack of n-3 LC-PUFA to supply human needs for a healthy diet must be considered. 

## 3. The Roles of Aquaculture

Considering the origin of n-3 LC-PUFA in the marine environment it is therefore no surprise that fish and seafood, traditionally from capture fisheries, have been the source of virtually all the EPA and DHA in our diet. It is well documented that capture fisheries around the world are either at or beyond their sustainable limits, plateauing at around 90 million tonnes per annum for the last decade or so and there is no prospect of this increasing in the future [26]. However, with an ever-growing global population, demand for fish and seafood has constantly increased and aquaculture has increasingly filled this demand with over half of all fish and seafood now being farmed [26]. However, there is a problem. While fish vary in the amount of n-3 LC-PUFA they can contain, depending largely upon whether oily or lean, they produce very little or none themselves, again dependent upon species. Therefore, essentially all the EPA and DHA they contain is derived from their diet, the natural prey organisms in wild-captured food fish or the manufactured feeds for farmed fish [2]. Consequently, the only way to ensure farmed fish contained high levels of EPA and DHA was to include these fatty acids in the feed and, until now, the only way to do this was to use large amounts of fish oil and fishmeal, themselves derived from marine fisheries that are similarly finite and limiting [26]. This, then, is the paradox. While aquaculture has increasingly become the major source of EPA and DHA for the burgeoning human population, it has also, at the same time, become the greatest consumer of the world’s available supply of EPA and DHA [27] and, consequently, the sector most proactive in ensuring adequate future supplies [28].

**Figure 2:**
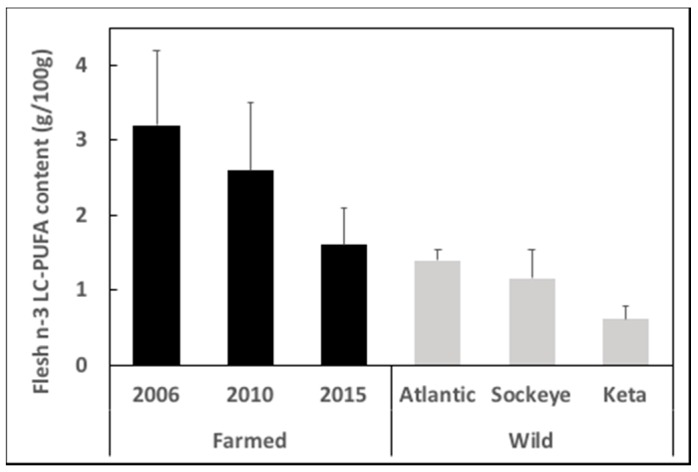
Content of n-3 long-chain polyunsaturated fatty acids (LC-PUFA) in flesh of Scottish farmed Atlantic salmon (*Salmo salar*) in 2006, 2010 and 2015, and in wild Atlantic and Pacific (Sockeye, *Oncorhynchus nerka* and Keta, *Oncorhynchus keta*) salmon. Data are presented as g n-3 LC-PUFA per 100 g flesh and are means ± SD and are taken from Sprague et al. [31,33,34].

The limitation in the supply of EPA and DHA has been an issue in aquaculture for over 20 years. Initially the response was dictated by market forces that drove up prices of fish oil and fishmeal, which naturally balanced demand to the existing supply. Thus, as aquaculture and the required feed volumes expanded, the limited amount of fishmeal and fish oil was spread thinner and thinner across the feeds, with fish oil being increasingly replaced by vegetable oils devoid of n-3 LC-PUFA, but often containing high levels of n-6 PUFA [29]. While this strategy has been very successful in both supporting the increasing demand for seafood and, at the same time, increasing the sustainability of feeds with lower dependence upon marine ingredients [30], the consequently lower levels of EPA and DHA in feeds was reflected in the farmed fish, impacting the nutritional quality of the farmed products (Figure 2) [31,32]. 

However, this is not only an impact on human consumers, because it also has potential consequences for the health of farmed fish themselves [35,36]. Thus, just as n-3 LC-PUFA are essential components of a healthy diet in humans, so they are in all vertebrates, including fish, and therefore replacing these in the diet with n-6 PUFA is actually having the same effect in fish as has happened in humans, with diets now having an imbalance of n-6 to n-3 fatty acids [2,37]. Therefore, it has long been appreciated that continued, progressive dilution of the levels of n-3 LC-PUFA in aquafeeds may impact upon the health of farmed fish, and so was an ultimately unsustainable strategy [2]. While, initially, considerable research effort went into mitigating the negative impacts on n-3 LC-PUFA levels of replacing fish oil and fishmeal in feeds with vegetable oils and plant proteins [29], recent research has focussed on finding more permanent solutions to the supply of EPA and DHA [2,9]. 

## 4. The Fundamental Solution

As EPA and DHA are naturally produced essentially only in the marine environment, that has been an area of focus for efforts to find new sources. Such initiatives include other unexploited fisheries, such as mesopelagic fish species, or fishing down the trophic levels, including zooplankton such as krill or copepods. These options have been the subject of other reviews [2,38] and further detailed review of these options is beyond the scope of this article. Suffice to say that, other than a relatively small commercial krill fishery that provides limited amounts of krill meal and oil, other marine options have, so far, not provided a solution to the problem of the supply of EPA and DHA [38]. Similarly, recycling activities such as extracting oils from the by-products of wild capture fisheries and aquaculture is already occurring, but is more an issue of efficiency in the use of existing, ultimately marine-derived, sources and not new supplies of n-3 LC-PUFA [2]. Therefore, while obviously desirable, recycling cannot be the solution to a problem that is only going to get bigger, i.e., the demand will constantly increase due to population growth, as increasing efficiency by reducing waste can only ever maximise the use of the existing, limited supplies.

All of the above represent further options for “harvesting” EPA and DHA, which is simply applying a 19/20th century approach. It is clear that we require entirely new (*de novo*) supplies of EPA and DHA and this, in turn, requires completely different approaches. In other words, we must generate the EPA and DHA that we require, rather than simply recycle it. One approach has been to apply modern technology to “farm” the marine organisms (i.e., the microalgae) that make the EPA and DHA in the first place. While feasible, this option has required advances in industrial technologies to enable scale-up of fermentation of heterotrophic species and/or bioreactor-like culture of phototrophic species [39]. A second approach has been to use 21st century molecular technologies to utilise the microalgal genes for the production of EPA and DHA in a well-established, efficient oil-producing platform, oilseed crops [40,41]. Crops already produce almost 200 million tonnes of vegetable oils annually with the only problem being that none of the plants can make the n-3 LC-PUFA, EPA and DHA, that we need. 

## 5. Alternative New Sources of EPA and DHA

Table 1 provides a brief summary overview of some potential new sources of EPA and DHA. Some of these novel sources are already commercially available products while others are in development and may well be available in the near future. Still others have been commercial products but appear to be no longer available, and so there has been some volatility in this rapidly developing and highly competitive market. The latter point also means that obtaining accurate, current data on the different alternative new sources can be challenging and, therefore, some of the data in Table 1 may also be subject to change. 

The purpose of Table 1 is simply to give the reader an appreciation of the range of potential new sources of EPA and DHA and how they differ in some key characteristics that may influence how they are used. In this context, there are essentially only two main options for *de novo* production of EPA and DHA, microalgae and genetically-modified oil producing organisms, particularly oilseed crops. The microalgal sources are currently largely derived from the production of Thraustochytrid species such as *Schizochytrium* and, occasionally, dinoflagellates like *Crypthecodinium cohnii*, that can be produced heterotrophically by fermentation. This technology is scalable but limited mainly by the capex required to produce very large biofermentor facilities [53]. A potential limitation with *Schizochytrium* and *Crypthecodinium* ssp. is that they generally produce only DHA and not EPA and, therefore, most of the microalgal products are only sources of DHA (Table 1).

However, the Veramaris^®^ venture is utilising a *Schizochytrium* strain that produces both EPA and DHA [54], and this is also being produced as an oil, which favours its inclusion in aquafeeds as it can be simply included as a direct substitute/replacement for the oils (fish and vegetable oil blends) currently used [55]. In contrast, the other microalgal products are all produced and marketed as algal biomasses that are generally around 50% lipid, with the other 50% being accounted for by varying proportions of protein, ash, and carbohydrate [56]. The precise nutrient composition of the algal biomass will influence its use in feeds. This is also the case with the yeast *Yarrowia lipolytica*, which was used as a biomass (approx. 50% lipid) when it was included in feeds for Atlantic salmon, *Salmo salar* [57,58].

As oils, the GM oilseed products have, like Veramaris^®^, the advantage of being able to be used in feeds as direct oil replacements. Production is also easily scalable as the infrastructure for the cultivation, harvest, processing, distribution, marketing and utilisation of vegetable oils is highly organised and well-established [59]. Depending upon the process of oil extraction it is possible that added value may be obtained by the production of seed meals containing residual n-3 LC-PUFA, which could be a commercial advantage supporting their use in animal feeds, within the normal limits for canola/camelina meals based on their other nutritional (and antinutritional) properties [60,61,62]. The GM oilseeds also provide a variety of fatty acid compositions including an EPA-only oil or several oils containing both EPA and DHA, from one containing high levels of both EPA + DHA, to oils that are predominantly EPA or DHA with lower amounts of the other n-3 LC-PUFA [48,63,64]. This offers the opportunity to utilise the oils in different ways.

## 6. Can these Products be Alternatives to Fish Oil in Aquaculture?

As fish oil has been our main source of EPA and DHA up until now, the above question may appear the obvious one to ask, however, not without some further explanation. The use of the word “alternatives” was deliberate as these products should be regarded as options to “supplement”, and not simply “replace” existing fish oil supplies. This is not a matter of semantics, while fish oil is undoubtedly finite on an annual/seasonal basis and, therefore, limited, it is not necessarily an inherently “unsustainable” product. Like all fisheries globally, if the fisheries supplying fishmeal and fish oil are managed effectively through regulation and control, and many are, there can be a sustainable harvest of this valuable commodity and there is no sense in not utilising this [65]. This is supported by the fact that the conversion ratio in the wild is around 10 kg of prey to 1 kg of food fish, whereas aquaculture is far more efficient and productive [66]. Furthermore, harvesting fish higher in the marine food web, such as cod, was shown to be far less efficient in providing marine nutrients for human consumption compared to harvesting lower trophic level species such as capelin (on which cod predate) and producing meal and oil for salmon production [67]. Thus, high trophic level predators may require many times more marine resources than lower trophic level fish, such as menhaden or anchovy used for reduction [65,68] and, hence, the importance of taking primary productivity into account when comparing the eco-efficiency of reduction fishing with fishing of higher trophic species for direct human consumption. 

Table 2 briefly summarises fish feeding trials using some potential new sources of EPA and DHA. This is not intended to be a comprehensive review of all trials involving microalgal or GM sources of n-3 LC-PUFA. Table 2 is restricted to the products listed in Table 1, or similar, related materials tested as part of the development process, and limited to published and/or publicly available data. Further details of studies on the use of microalgal products, largely biomasses, in on-growing feeds, as opposed to larval feeds, was briefly reviewed recently [9]. In summary, there have been several trials with a variety of algal biomasses in recent years with generally positive results, although there are two caveats. Firstly, biomasses cannot directly replace the oil component of fish feeds due to the presence of protein and, especially carbohydrate (largely non-starch polysaccharide, NSP) and this can limit the total amount that can be incorporated into optimally balanced feeds without negative impacts. 

Secondly, the biomasses only supply DHA, which has prompted considerable interest and attention on DHA and EPA individually, and their relative value as essential fatty acids (EFA) [85]. While DHA is better retained than EPA, this probably does not reflect function, but rather is simply due to the oxidation of DHA for energy being more complicated, requiring the involvement of peroxisomes [2,86]. In addition, EPA and DHA differ in some fundamental antioxidant and membrane effects [87] and, in this respect, DHA has specific key roles in neural (brain and eye) development and function that EPA does not [88]. However, when it comes to metabolic and physiological control and regulation, EPA and DHA both have important and different, albeit overlapping, roles. A detailed examination and appreciation of their respective roles in controlling metabolism (e.g., as ligands for transcription factors etc.) and also in inflammation, the key fundamental step in the response to any injury or infection and the immune system, is outwith the scope of this review, and is covered elsewhere [6,7,89,90]. Suffice to say that it is likely that an ideal new n-3 LC-PUFA source would supply both EPA and DHA.

Peer-reviewed data on trials investigating the use of oils from genetically engineered oilseed crops is fragmentary, being quite comprehensive in the case of GM Camelina, but more elusive in others. An early study showed that oil derived from an GM oilseed crop could be used in aquafeeds without any apparent negative effects related to “GM” [91]. While this was a stearidonic (18:4n-3)-containing oil from genetically engineered soybean, and so not a direct source of EPA or DHA, it at least proved the general concept. While information on trials involving oils derived from GM canola is sparse, recent conference reports indicate that they have both been used successfully in trials generally replacing the fish oil component in feeds for Atlantic salmon [83,84]. In contrast, studies investigating the oils derived from genetically-engineered *Camelina sativa*, including both EPA-only and EPA+DHA versions, have been extensively reported both in Atlantic salmon [48,49,63,80,82] and sea bream, *Sparus aurata* [81]. In all these studies, the modified camelina oils have been used to replace both fish oil and vegetable oil, showing that they can directly replace all the added oil in feeds without negative impacts on fish growth or health. The most recent study showed that replacing both fish oil and rapeseed oil with a camelina oil containing > 25% n-3 LC-PUFA doubled the EPA+DHA level in the flesh of the salmon compared to fish fed the current commercial standard feed [49], suggesting that this oil could restore levels in harvest-size salmon to those last seen more than a decade ago before the development of sustainable feeds with high replacement of marine ingredients [31]. 

All the above confirms that the new sources of EPA and DHA have the potential to increase n-3 LC-PUFA levels in salmon and other farmed fish with no major negative impacts on fish growth, feed efficiency and health. Thus, they can be real, practical alternatives to fish oil, not only in supplying energy and satisfying EFA requirements of the fish, but also, most importantly, increasing levels of EPA and/or DHA to pass on to human consumers ensuring farmed fish retain their position as key components of a balanced, healthy diet. 

## 7. Current Use in Commercial Aquafeeds and other Commercial Products

Algal biomasses are already being used in commercial aquafeeds. ForPlus™ is currently being included in the formulation of a number of feeds produced by Alltech^®^ Coppens including for salmonids (e.g., the fishmeal and fish-oil-free “Neogreen” feed) and marine species, as well as broodstock diets for freshwater species, although the exact amounts incorporated in the feeds are unknown [92]. It is also unclear if this will continue in the future with the closure in January 2018 of the Alltech US microalgae facility in Winchester, Kentucky [93]. The joint venture between Terravia and Bunge resulted in a new large fermentation facility being established at a Bunge site in Brazil processing sugarcane. This close association of algal production with the fermentation feedstock increased the production volume of AlgaPrime™ DHA enabling an agreement for Terravia/Bunge (now Corbion) to supply this product to the BioMar Group for inclusion in fish feed [94]. A similar strategy to the above has been implemented by Veramaris^®^ with a new fermentation facility being built in Nebraska to use US corn syrup as fermentation feedstock, with initial production capacity upon commercial release in 2019 projected to meet up to 15% of the total current annual demand for EPA and DHA by the global salmon aquaculture industry based on current inclusion levels [54]. 

The development of GM *Y. lipolytica* as a source of EPA by DuPont resulted in two commercial products including New Harvest^TM^ EPA, an encapsulated oil for direct human consumption, and Verlasso^®^ salmon, produced by AquaChile in 2016 using *Y. lipolytica* biomass in the feed [95]. However, Verlasso^®^ salmon are now being produced using microalgae, rather than GM yeast biomass, although the precise source and nature of the microalgal product is not known. While none of the GM oilseed oils are yet fully commercialised both the groups involved in developing GM canola are actively seeking approval for planting of the elite canola events they wish to commercialise through the publication in the USA of the USDA Animal and Plant Health Inspection Service (APHIS) “Petition for non-regulatory status” [50,51]. In addition, Nuseed have announced that two commercial products are to be marketed including Aquaterra™ for aquaculture, and Nutriterra™ for human consumption [96]. Similarly, very recently Cargill announced the launch of Latitude™, their “a sustainable, plant-based alternative source of omega-3 for fish feed applications” [97].

## 8. The Future

The above has demonstrated that the new sources can, to varying degrees depending upon precise fatty acid composition, supply EPA and/or DHA and, therefore, supplement the existing supplies of fish oil that will always be finite and limiting. But what exactly will be the future for all these varied products? How will they be used and how will their different origins and compositions affect their use? While this article has focused on the different biological and chemical characteristics of the new sources, they also vary in the way their use could be impacted by socio-economic and socio-political issues. While the microalgal products may have price implications associated with the relatively high cost of production [53,93], GM-derived oils have issues associated with consumer perception and acceptance in some countries, especially in the EU [98,99], as well as the hurdle of regulatory approval. Furthermore, the gap between supply and demand was based on the calculated ‘physiological demand’, which is different to ‘commercial demand’. The latter currently includes demand from the direct human consumption (capsule) market, as well as potential future demand from other animal feed (poultry and pig) sectors, but the main driver will likely continue to be the aquaculture sector. However, even within aquaculture, the use of the different new sources will not only be dependent upon biological and technical factors of feed formulation and manufacture, but also the overall aim and ambition of the sector, which is currently not clear and likely to be driven by market forces dependent upon availability of supply and cost.

### 8.1. Use in Aquafeeds to Maintain Fish Growth and Health

The least ambitious aim would be to ensure aquafeeds satisfy the essential physiological requirements of the fish itself, which minimally is the level required to prevent deficiency signs, but optimally the level to ensure maximum growth and good health [2]. This would parallel the current situation with essential amino acids (EAA). As certain EAA are deficient in many plant meals, feeds are generally supplemented with small amounts of crystalline EAA (e.g., lysine and methionine) to ensure requirements are met [60]. In the same way, high proportions of vegetable oils devoid of EPA and DHA are used in more sustainable feed formulations and, while the feeds still satisfy minimum requirements to prevent EFA deficiency, they may now be approaching sub-optimal for fish health. This is exactly the situation faced by many human populations where instances of EFA deficiency are rare or virtually unknown, but many/most have dietary levels of n-3 LC-PUFA and, especially EPA and DHA, that are below dietary recommendations for optimal health [25]. In terms of EPA and DHA content, microalgal oil (e.g., Veramaris^®^), is the closest EFA equivalent to crystalline EAA and so it could be used in relatively small amounts to supplement current formulations with sufficient EPA+DHA to ensure optimum fish health. Of course, while basic EFA requirement levels may be known, the level of EPA+DHA required to ensure maximum growth and optimal health in fish fed modern, high performance (energy) feeds has arguably not been determined empirically for any fish species [85]. Furthermore, a strategy focussing primarily on the requirements for fish health, and using only relatively small amounts of the new EPA and DHA sources to replace some or all of the fish oil currently used in feeds, would not tackle the gap between supply and demand for human consumers.

### 8.2. Use in Aquafeeds to Support Human Health

While the initial priority may be to supply sufficient dietary EPA and DHA to ensure fish health and at least maintain current levels in farmed products [84], the ultimate goal should be to significantly increase dietary levels so that farmed fish can deliver similar or higher levels of n-3 LC-PUFA than wild fish. This will establish farmed fish as the best way by far for people to supplement their diets with “omega-3” and confirm fish as key components of a healthy balanced diet. 

Of course, ensuring that farmed fish can deliver high levels of EPA+DHA to human consumers would require much higher levels to be included in the fish feed. This would mean using the new sources not to replace some of the fish oil, but to replace the vegetable oil currently used in feeds. How to implement this strategy would depend upon the fish species. Fish with low flesh fat content, like many marine species, will require less dietary EPA+DHA to increase the proportions of these fatty acids in the flesh, which is good, but they will not deliver the same ‘dose’ of EPA+DHA to human consumers that an oily species like salmon can. However, for farmed salmon to deliver this high dose it requires high levels of EPA+DHA in the feed. This will not be possible with microalgal biomasses that cannot be incorporated into the feeds of salmonids, or marine fish, at the required high levels due to the presence in the biomasses of substantial amounts of carbohydrates that, irrespective of the exact type of carbohydrate, have very low nutritional value in carnivorous fish species with limited ability to utilise dietary carbohydrate of any type [100]. However, this may not be exactly the case in lower trophic level species with more omnivorous or herbivorous feeding habits [100]. 

### 8.3. Different Products, Different Niches?

The above sections discussed potential aims for using the new sources of EPA and DHA and how different products could sit within these two main options. The following further explores the options for using the new sources of EPA and DHA, specifically matching key characteristics of the varied products with specific niche sectors within aquaculture. 

**Figure 3:**
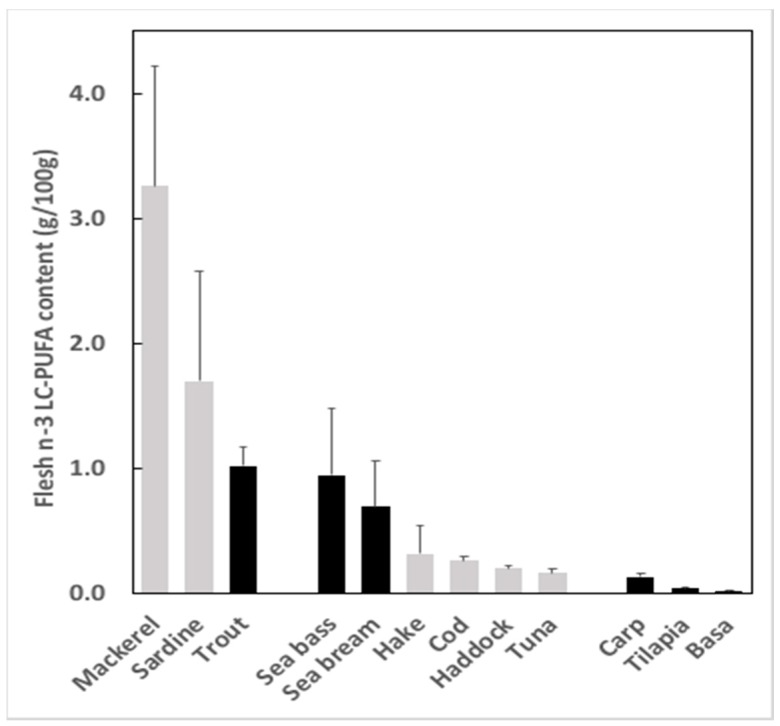
Content of n-3 long-chain polyunsaturated fatty acids (LC-PUFA) in flesh of fish species common in seafood markets including oily species, Atlantic mackerel (*Scomber scombrus*), sardine (*Sardina pilchardus*) and rainbow trout (*Oncorhynchus mykiss*); marine species, Atlantic cod (*Gadus morhua*), Atlantic haddock (*Melanogrammus aeglefinus*), European hake (*Merluccius merluccius*), European sea bass (*Dicentrarchus labrax*), gilthead sea bream (*Sparus aurata*) and Yellowfin tuna (*Thunnus albacares*); freshwater species, common carp (*Cyprinus carpio*), basa (*Pangasius bocourti*) and tilapia (*Oreochromis niloticus*). Data are presented as g n-3 LC-PUFA per 100 g flesh and are means ± SD and are taken from Sprague et al. [33,34] and Sprague (unpublished data). Bar colour denotes whether the analysis was of farmed (black) or wild (grey) samples.

As alluded to above, microalgal biomasses can be included in feeds for carnivorous species to only a limited extent, at least partly due to the carbohydrate content that is relatively high, possibly 10–30% depending upon species albeit details of carbohydrate compositions are difficult to obtain for any the biomasses or *Schizochytrium*/thraustochytrid species generally [101]. Most, if not all, of the carbohydrate is assumed to be NSP, likely associated with the cell wall that, in addition to being indigestible may further impact digestion of other nutrients, and so generally only 5–6% of algal biomasses can be incorporated in feeds for salmon without compromising growth [56]. Fish species at lower trophic levels, specifically herbivorous/omnivorous fish such as carps, tilapia and pangasius, may have (slightly) better capacity to digest cell walls, if they have β-glucan components, due to the possible presence in the intestine of enzyme activities (e.g., cellulases), whether of endogenous or, more likely, microbial origin [100]. Therefore, if these species were more tolerant of algal biomasses, and able to better digest biomass, increasing bioavailability of protein, lipid and minerals [102], this could enable higher inclusion levels in feeds, and so could arguably be the niche market for these products [103]. However, these species are generally not particularly high value fish, which makes the economics of enhancing EPA + DHA levels in these species more difficult [93]. Similarly, they are not high fat fish and so can only provide a limited ‘dose’ of EPA and DHA to consumers (Figure 3) [33]. However, these species are produced in huge tonnages and are important components of human diets in many parts of the world, especially Asia and Africa, and so improvement of their nutritional value through increased levels of DHA could benefit a very large number of people, many of whom have low levels of n-3 LC-PUFA in blood [22]. 

The high EPA+DHA oils such as the microalgal oil, Veramaris^®^, and the oil from genetically engineered Camelina, would be ideal for improving the n-3 LC-PUFA content of oily species such as the salmonids, especially salmon, where flesh fat (muscle lipid) is dominated by triacylgycerol whose fatty acid composition closely reflects the fatty acid composition of the diet [104]. This is the most efficient way of passing the high levels of EPA and DHA in these products on to human consumers via farmed fish. With the microalgal oil, suitably high dietary levels of EPA+DHA would not necessarily require the oil to be included at 100% of the added dietary oil. Due to the high proportion of n-3 LC-PUFA in the microalgal oil, near twice the level found in southern hemisphere fish oil, a 50:50 blend with rapeseed oil could probably restore EPA+DHA levels in farmed salmon to that of a decade ago before the development of sustainable feeds [31]. An alternative would be to just use one oil at 100% of the added oil and this is precisely what the GM Camelina crop was designed to provide. The modified camelina seed oil contains 20–25% n-3 LC-PUFA and, when used to replace both FO and rapeseed oil, the level of n-3 LC-PUFA in flesh was doubled, suggesting that it could restore levels in harvest-size fish to those of more than a decade ago, prior to the development of sustainable feeds [49,63]. 

In contrast, oils with lower proportions of total n-3 LC-PUFA may be less than ideal for oily species like salmon in which flesh fatty acid composition closely reflects dietary fatty acid compositions. However, this may be less important in species that have lower levels of lipid in their flesh and where a greater proportion of EPA and DHA in flesh lipid will be present in phospholipids rather than in triacylglycerol [104]. Thus, the oils from other GM plants, which contain around 10–12% total n-3 LC-PUFA could arguably be best used in feeds for non-oily marine fish such as sea bream and sea bass, as well as crustaceans (shrimp and prawns). These oils are also characterised by being either predominantly EPA (BASF/Cargill) or DHA (Nuseed/CSIRO) that, as discussed briefly above, both have important but different metabolic and physiological roles. Assuming that some balance between these two key n-3 LC-PUFA is desirable, albeit precisely what the optimum balance might be is unknown, a blend of these two oils may be more beneficial than either individually, with a 1:1 blend probably a reasonable starting point.

The above discussion simply aligns biochemical/nutrient composition data of the various new sources of n-3 LC-PUFA with biological/physiological characteristics of aquaculture species and suggests some potential matches. While these considerations could clearly aid the efficient utilisation of EPA and DHA in the human food chain, it is acknowledged that market forces underpinned by many other economic and socio- and geo-political factors will be equally important drivers for the new n-3 LC-PUFA value chain. 

### 8.4. Non-Aquaculture Uses

As long as there is a limited supply, aquaculture will remain the ideal delivery system for n-3 LC-PUFA to human consumers. As well as fish and seafood being well appreciated by consumers as the traditional way to obtain these key nutrients, aquaculture production is generally far more efficient than any terrestrial animal production [105]. Thus, highly efficient species such as Atlantic salmon [106] feed conversion ratio (FCR) of around 1.1 to 1.2 in commercial production, compared to ~2 for poultry, ~3 for pigs, and > 6 for lamb and beef [105]. In addition, salmon show higher protein, energy and n-3 LC-PUFA retentions, and harvest and edible yields than terrestrial meat production [105]. However, there can be further options if there were sufficient supplies of n-3 LC-PUFA to support other animal feed uses as well as direct human consumption. Although not such an efficient way of utilising EPA and DHA, supplementation of feeds for monogastric animals like pigs, and poultry (broilers and layers), could increase n-3 LC-PUFA levels in pork [107], chicken [108] and eggs [109] as in the past when feeds for these animals often contained fishmeal [110]. Supplementation of feeds for ruminants such as cattle or sheep will only be sensible if good encapsulation methods were available to protect the fatty acids in the rumen and prevent loss by biohydrogenation [111]. Supplementation of other food groups with EPA and DHA is also a possible option especially after microencapsulation, and has been used with milk, cheese, yoghurt, bread and juice [112,113]. The more widespread availability of EPA and DHA in food may appear a long-term goal but one that has historically received attention in relation to human dietary requirements [114]. In 2011, the Omega-3 summit recommended a “Dietary intake of > 1000 mg of n-3 LC-PUFA was needed if consuming a western-type diet”, which was indicating that current dietary recommendations [14] may be insufficient to ensure a balanced healthy diet [115]. This was further discussed at the recent 2018 Omega-3 Summit conference that held four key debate sessions, “Strategies for Increased use of n-3 LC-PUFA for Improving Health”, “Matching Omega-3 Requirements with Omega-3 Supplies”, “Strategies for the Industry to Improve the Global Intake of n-3 LC-PUFA” and “Strategies for Food and Food Ingredients Industry to Deliver n-3 LC-PUFA to Consumers Worldwide in a Sustainable Way” [116]. 

## 9. Conclusions

The present review has highlighted the fundamental problem of inadequate supplies of n-3 LC-PUFA to satisfy human physiological requirements, briefly summarised the potential new sources of these key nutrients, and provided some analysis of how these new products could be used effectively. The overall conclusion is that all the new sources can have important roles to play in helping to support healthy, balanced diets for people. The variety of new sources of EPA and DHA coming to market will be highly beneficial as they can be targeted to different uses and, as they all find their niche markets, this could help to draw down prices making their widespread use increasingly feasible.

## Figures and Tables

**Table 1:** Summary of the origins and compositions of some potential new sources of EPA and DHA.

				Composition ^a^					
							Total n-3 LC-PUFA		
Product	Development Partners	Source	Type	Lipid Content ^b^	EPA ^c^	DHA ^c^	% of TFA ^d^	% of Product	Reference
AlgaPrime™ DHA	Corbion (TerraVia/Bunge) ^e^	Microalgae	Algal biomass	60	0	48	48	28	[42]
DHAgold™	DSM Nutritional Products	Microalgae	Algal biomass	49	1.0	44.4	45.8	22.5	[43]
DHA Natur™	ADM Animal Nutrition	Microalgae	Algal biomass	50–60	0.25	34	34.3	17.2–20.6	[44]
ForPlus™	Alltech Coppens ^f^	Microalgae	Algal biomass	61	0.3	29	29.3	17.9	[45]
Nymega™	Heliae Development ^g^	Microalgae	Algal biomass	65	~0.1	20	~31	21	[46]
Veramaris^®^ Oil	Veramaris ^h^	Microalgae	Oil	100	~16	~34	~54	~54	[47]
*Camelina sativa*	Rothamsted Research/UoS	GM camelina	Oil	100	20	0	24	24	[48]
*Camelina sativa*	Rothamsted Research/UoS	GM camelina	Oil	100	9	11	28	28	[49]
Latitude™	BASF/Cargill	GM canola	Oil	100	7	1	12	12	[50]
Aquaterra™/Nutriterra™ ^i^	CSIRO/Nuseed/GRDC	GM canola	Oil	100	0.5	10	12	12	[51]
*Yarrowia lipolytica* ^j^	DuPont	GM yeast	Yeast biomass	~50	~50	0	50	25	[52]

^a^ Compositional data were extracted or extrapolated from various sources and, in some cases, may differ in detail from final commercialised products. ^b^ Percentage of product; ^c^ Percentage of total fatty acids (TFA); ^d^ Includes 20:4n-3 and 22:5n-3 (if any); ^e^ Initially developed by Terravia Holdings (formerly Solazyme) and to be commercially produced and marketed in joint venture with Bunge. Acquired by Corbion after Terravia filed for bankruptcy with subsequent buyout of Bunge; ^f^ Initially developed by Alltech and, after acquiring Coppens International feed company, was included in many of Alltech Coppens aquafeeds although this may not continue in the future with the closure of the microalgal facility in the USA; ^g^ Initially developed by Heliae Development LLC and distributed in partnership with Syndel Laboratories Ltd.; ^h^ Initially developed by DSM and to be produced and marketed by Veramaris^®^, a 50:50 joint venture with Evonik; ^i^ Oil from GM canola to be marketed as Aquaterra™ and Nutriterra™ for aquaculture and human consumption, respectively; ^j^ An oil from GM *Y.lipolytica* was once marketed in capsule form as New Harvest™ EPA for human consumption. Compositional data extracted or extrapolated from the publications or publicly available materials cited in last column. ADM, Archer Daniels Midland Company; CSIRO, Commonwealth Scientific and Industrial Research Organisation; GRDC, Grains Research and Development Corporation; UoS, University of Stirling.

**Table 2:** Studies using the products listed in Table 1, or similar, related materials tested as part of the development process, as ingredients in feeds for fish ^a^.

Product	Species	Replacing	Inclusion Levels	Reference
AlgaPrime™ DHA biomass ^b^	N/A			
DHAgold™ biomass	Cobia (*Rachycentron canadum*)	FO and soybean oil	1.5, 2.2, 3.0 and 4.3% of diet ^c^	[69]
	Atlantic salmon (*Salmo salar*)	FO, FM and plant meals	5.5 and 11% of diet ^c^	[70]
	Rainbow trout (*Oncorhynchus mykiss*)	Corn oil and wheat flour	3, 6 and 9% of diet	[71]
DHA Natur™ biomass	Shrimp (*Litopenaeus vannamei*)	FO and VO, soybean meal and wheat starch	1.3 and 5.0% of diet	[72]
ForPlus™ biomass	Drum (*Totoaba macdonaldi*)	Corn starch	0.2 and 0.6% of diet	[73]
	Atlantic salmon	FO, horse beans, maize gluten	1, 6 and 15% of diet	[74]
	Atlantic salmon	FO and FM	2.5 and 5.0% of diet	[45]
	Shrimp (*Litopenaeus vannamei*)	FO and wheat flour	0.6, 1.2, 1.8, 2.3 and 3.5% of diet	[75]
	Longfin yellowtail (*Seriola rivoliana*)	FM	5% of diet	[76]
	Giant grouper (*Epinephelus lanceolatus*)	FO, FM and squid	10, 15 and 18% of diet	[77]
	Red drum (*Sciaenops ocellatus*)	FO, SPC and FM	1.3, 2.7, 4.0, 5.4, 6.7 and 9.9% of diet ^d^	[78]
Veramaris^®^ oil	Atlantic salmon	FO	25, 50, 75 and 100% of FO ^e^	[55]
		FO and FM	2–4 and 8–15% of diet	[79]
*Camelina sativa* EPA oil	Atlantic salmon	FO and RO	100% of added oil	[48,80]
	Sea bream (*Sparus aurata*)	FO and RO	100% of added oil	[81]
*Camelina sativa* EPA+DHA oil	Atlantic salmon	FO and RO	100% of added oil	[49,63,82]
	Sea bream	FO and RO	100% of added oil	[81]
Latitude™	Atlantic salmon	FO	100% of added FO ^f^	[83]
Aquaterra™	Atlantic salmon	FO		[84]
*Yarrowia lipolytica* biomass	Atlantic salmon	FM, wheat meal and RO	10–30% of diet	[57,58]

^a^ Limited to studies that have been published or reported publicly (e.g., open conferences etc.). Many more studies have likely been performed in “in-house” research and development programmes. ^b^ N/A, None available. No published studies could be clearly identified as specifically testing AlgaPrime™ DHA; ^c^ AquaGrowGold^®^; ^d^ SP1 included along with Spirulina meal at a ratio of ~1:4.2; ^e^ A second study replaced 25, 50 and 100% of EPA+DHA of FO; ^f^ Two diets with amount of GM Canola oil added to supply equal amounts of either EPA+DHA or n-3 LC-PUFA (EPA+DHA+DPA). FM, fishmeal; FO, fish oil; RO, rapeseed oil; SPC, soy protein concentrate; VO, vegetable oils.
